# Essential role of community health workers in promoting oral health in Africa

**DOI:** 10.4102/jphia.v16i1.782

**Published:** 2025-06-03

**Authors:** Morenike O. Folayan, Ahmed Bhayat, Nicaise Ndembi, Adeyinka G. Ishola, Maha El Tantawi

**Affiliations:** 1 Department of Child Dental Health, Faculty of Dentistry, Obafemi Awolowo University, Ile-Ife, Nigeria; 2 Department of Community Dentistry, University of Pretoria, Pretoria, South Africa; 3 Director General’s Office, International Vaccine Institute (IVI), Africa Regional Office, Kigali, Rwanda; 4 Division of Epidemiology and Prevention, Institute of Human Virology, University of Maryland School of Medicine, Baltimore, United States of America; 5 Africa Centres for Disease Control and Prevention, Addis Ababa, Ethiopia; 6 Department of Mental Health and Psychiatric Nursing, Faculty of Nursing, University of Ibadan, Ibadan, Nigeria; 7 Department of Mental Health and Psychiatric Nursing, Faculty of Nursing Sciences, University of Ilesa, Ilesa, Nigeria; 8 Department of Pediatric Dentistry and Dental Public Health, Faculty of Dentistry, Alexandria University, Alexandria, Egypt

**Keywords:** health promotion, rural communities, public health, education and awareness, health systems, preventive care, access to care, training and capacity-building, integration into health systems, sustainable health practices

## Abstract

Oral health remains a critical yet often overlooked aspect of overall health in Africa, where a significant burden of oral diseases is evident. The integration of community health workers (CHWs) into oral health promotion strategies presents a unique opportunity to address both human resource and educational challenges, particularly in underserved communities. This study explores the pivotal role CHWs can play in enhancing oral health outcomes across various African contexts. By providing basic dental care, education and facilitating access to professional services, CHWs contribute to the prevention and early detection of oral diseases. The research draws on case studies, programme evaluations and field reports to highlight the effectiveness of CHW-led initiatives. The findings underscored the need for increased investment in CHW training and support as a sustainable approach to improving oral health in Africa. It highlights the role of the Africa Centres for Disease Control and Prevention in promoting oral health in the mandate of CHWs, identifies the challenges it may face in playing this role and proffers solutions including promoting the development of oral health policies and plans by the Africa Union Member States as a critical first and feasible step. The study concludes by identifying the need for a comprehensive assessment of the status of integration of oral health into CHW programmes in Africa to help the Africa Centres for Disease Control and Prevention identify gaps for strategic actions. This article offers the first comprehensive exploration of the potential for formally integrating CHWs into oral health promotion strategies across Africa. By mapping existing interventions, evaluating their effectiveness, and identifying policy and structural challenges, the study provides critical insights into how CHWs can bridge gaps in access to oral healthcare, particularly in underserved populations. It highlights the strategic role of the Africa CDC in advancing oral health through CHW-led initiatives and calls for standardised training, policy support, and system integration. The article aligns with the *Journal of Public Health in Africa*’s scope by addressing health systems strengthening and universal health coverage in the African context through an underutilised yet scalable workforce.

## Introduction

The ‘Community Health Worker (CHW) Programmes Policy Brief’ from the WHO African Region emphasises the expansion of CHW programmes to strengthen primary healthcare (PHC) and achieve universal health coverage (UHC).^1^ Community Health Workers, often local community members, play a crucial role in improving access to healthcare, especially in regions with limited access to health systems, by reaching underserved populations and providing culturally appropriate care.^2^ Their importance is growing because of the need to mitigate challenges such as climate change, food insecurity, disease outbreaks, migration and conflict through active community engagement.^3,4^

While CHWs have the potential to positively impact oral healthcare delivery in Africa, there is currently no strong evidence of the effectiveness of institutional CHW programmes for oral health. Oral diseases are often overlooked as a major public health issue in Africa despite the continent’s high disease burden. Although the prevalence of oral diseases may not exceed that of other regions, Africa’s large population amplifies the number of those affected.^5^ With a dentist-to-population ratio of approximately 1:22 010, and only about 66 360 dentists serving 1.5 billion people, the continent faces significant unmet treatment needs.^6^ Furthermore, expenditure on oral care in Africa is the lowest globally, predominantly financed out-of-pocket,^7^ adding to the financial burden on affected populations and their ability to seek dental care.

Community health workers can play a crucial role in supporting oral health in Africa by educating communities on oral hygiene, promoting regular dental check-ups and providing preventive services such as fluoride application and dietary counselling to reduce sugar intake. They can identify early signs of oral diseases, refer patients for further treatment and help prevent the progression of serious conditions.^8^ Involving CHWs in oral healthcare can increase referrals for advanced oral disease management from 63% to 84%.^9^ Community health workers’ services are often preferred over formal dental services in African communities because of their proximity and cost-effectiveness, resulting in higher satisfaction.^9^ By serving as educators, guiding individuals through healthcare systems and providing support to healthcare recipients, they can effectively bridge the existing gaps in access to dental care.^2^

Training CHWs to deliver basic oral care can also expand the dental workforce, especially in rural, marginalised and indigenous communities.^10^ These populations typically have higher rates of dental issues, fewer teeth, less health literacy and poorer access to care.^11^ Community health workers can address the shortage of dental professionals and inadequate health infrastructure, ultimately improving oral health outcomes, reducing the high disease burden and contributing to the resilience of oral healthcare systems in Africa.^12^ Their involvement supports UHC and can enhance public health across the continent.^13^

## Mapping the evidence on community health workers in Africa

Despite the vital role that CHWs play, there is a lack of consolidated evidence on the breadth and impact of CHW interventions across African countries. This gap in evidence underscores the need for a comprehensive review to better understand the role and effectiveness of CHWs in improving health outcomes and strengthening health systems in the region. To provide valuable insights into the effectiveness of CHW interventions and inform future strategies to enhance their contributions to public health in Africa, a mapping of primary studies conducted on CHWs in African countries that evaluated the impact of oral health interventions led by CHWs on oral health outcomes in African populations was undertaken.

Studies were included if they reported on the impact of the work of CHWs in African countries. The interventions examined had to be related to CHW activities, and the studies needed to report oral health outcomes such as oral health knowledge, behaviours or clinical outcomes, or programme evaluations. The studies had to be published between 2000 and 2024. Studies were excluded if they provided programme descriptions without reporting outcomes. In addition, they were also excluded if they were not published in English or French.

Search terms were designed to combine concepts related to CHWs, Africa, and primary studies by using Boolean operators (AND, OR). The search terms included phrases such as ‘community health worker*’, ‘CHWs’, ‘village health worker*’ and ‘lay health worker*’, combined with terms such as ‘Africa*’, ‘sub-Saharan Africa*’ and specific 54 countries in Africa. Grey literature from organisations such as the World Health Organization (WHO) and African ministries of health was also included. The references of the eligible publications were also studied to identify other possible eligible publications.

The search was conducted across multiple databases to ensure comprehensive coverage of the literature. This multi-database approach aims to capture a wide range of primary studies, ensuring a thorough and inclusive review of the available evidence. The databases searched include PubMed/MEDLINE, which contained 37 relevant records, Africa-Wide Information (EBSCOHost), which had seven records and African Journals Online (AJOL), which contained no records. Of the 44 records, 36 abstracts were excluded. After the review of the eight full articles, only three reported on the impact of CHW activities. The details of the three publications are in Table 1, focussing on different CHW types, study designs and outcomes.

**TABLE 1:** Characteristics of the publications on the impact of community health workers’ interventions on oral health in communities in Africa.

Author (year of publication)	Country; region in Africa	Study objective	Type of CHW involved in study	Sample size	Study design	Result of studies
Jamieson; 2006^14^	Uganda; East Africa	NA	Community women groups	1874	Quasi-experimental	After 1.5 years of the programme’s inception, community awareness of the scientific causes and alternatives to *ebino* extractions increased (as gauged by follow-up focus group discussion), and the number of hospital admissions for traditional tooth extraction complications decreased
Naidoo and Mbia; 2011^15^	Cameroon; Central Africa	Investigate the knowledge and practices of traditional healers (THs) regarding tooth extraction and the management of its complications	Traditional healers	16	Cross-sectional	The healers used herbs for pain and bleeding control and 62.5% referred complex cases to dentists. Among the 150 patients, 93.3% were satisfied, and 95.3% reported no post-extraction issue
Agbor and Naidoo; 2011^16^	Cameroon; Central Africa	Determine the oral care knowledge and practices of THs regarding oral care delivery in urban and rural areas of Bui Division of Cameroon, the cost of treatment and reasons why people visit TH	Traditional healers	21	Cross-sectional	Oral treatment includes pain management, removal of worms from infected teeth and tooth extractions. Only 6% of patients who visited THs were referred to dentists. Yet 67% of the 52 patients reported satisfaction. Affordability drives TH visits (69%), as their treatment costs were around $5.00 compared with $50.00 for conventional care

Note: Please see the full reference list of the article Folayan MO, Bhayat A, Ndembi N, Ishola AG, El Tantawi M. Essential role of community health workers in promoting oral health in Africa. J Public Health Africa. 2025;16(1), a782. https://doi.org/10.4102/jphia.v16i1.782, for more information.

CHW, community health workers.

The study by Jamieson^14^ in Uganda evaluated a community intervention involving women’s groups by using a quasi-experimental design. It demonstrated increased awareness of scientific explanations for traditional tooth extraction practices and a reduction in hospital admissions for complications from ‘ebino’ extractions. This suggests that targeted community education programmes can positively influence oral health awareness and behaviours.

Naidoo and Mbia^15^ in Cameroon used a cross-sectional design to assess the knowledge and practices of traditional healers (THs) regarding tooth extractions. The findings showed that all healers used herbal remedies for pain and bleeding control, with 62.5% referring complex cases to dentists. Patient satisfaction was notably high (93.3%) and 95.3% reported no post-extraction complications. This highlights the significant role that THs play in oral healthcare despite limited formal training.

Agbor and Naidoo^16^ expanded on the previous study by investigating oral health knowledge, treatment practices and cost considerations among THs in urban and rural Cameroon. They found that THs managed pain, extracted teeth and even claimed to remove ‘worms’ from infected teeth. However, referrals to dentists were rare (6%), although patient satisfaction remained high (67%). The study also emphasised economic factors, with TH treatment costs averaging $5.00 compared with $50.00 for conventional care, making THs the more affordable option.

Together, these studies underscore the complex interplay between traditional and formal oral healthcare systems in Africa. While CHW interventions can enhance awareness and reduce harmful practices, traditional healers remain a crucial, affordable alternative for many, despite limited referrals and reliance on herbal remedies.

We also identified a mirage of formal and informal workers who play roles in oral health promotion and service provision. These included school teachers and peers.^7,12^ There are opportunities to integrate oral health promotion into the ongoing health promotion activities of CHWs as many meet clients with oral health problems, which they have limited capacity to address.^17,18^ Training can help build this capacity not only to improve the competency to conduct oral health education and screen for oral diseases,^19,20^ but also to provide basic oral healthcare like atraumatic restorative treatment.^21^

## Challenges community health workers face in delivering oral health services in Africa

### Siloed programmes

Community health worker programmes often operate in isolation, with fragmented coordination, leading to gaps in care continuity and communication issues.^22^ This lack of integration with the PHC structure prevents seamless information flow and comprehensive healthcare. Without being integrated into the formal healthcare system, CHWs receive less supervision and support, leading to inconsistent care quality and reduced impact.

Existing CHW efforts in oral health are limited to project-based initiatives rather than integrated national programmes.^23^ Examples include training school teachers in Zimbabwe on dental care,^24^ CHWs in Kenya to recognise human immunodeficiency virus (HIV)-related oral lesions^25^ and community oral health workers in Gambia to provide atraumatic restorative treatment.^19^ In South Africa, health promotion officers were trained to integrate oral health literacy and THs were educated to diagnose and treat common oral conditions.^8^ In Nigeria, CHWs were trained to deliver oral health education to nursing mothers.^26^ These projects highlight the potential of CHWs in delivering oral healthcare, but national integrated programmes are still needed. A 2022 systematic review identified Cameroon, Gambia, Kenya, South Africa, Uganda and Zimbabwe as countries utilising CHWs for primary oral healthcare.^27^ However, these CHWs vary widely in their years of training, qualifications and roles,^28^ indicating a need for more structured and uniform programmes across the continent.

### Policy and governance

The literature shows a lack of clear policies and governance structures to support CHW programmes, which leads to unclear roles, responsibilities and career development paths for CHWs. Despite global and national commitments, reflected in the global strategy on human resources for health by 2030^22^ and the WHO guidelines,^29^ many African countries still struggle to establish governance frameworks that clearly define CHWs’ roles and responsibilities. This lack of clarity creates inefficiencies and overlapping responsibilities and hampers the integration of CHWs into national health systems.

The current CHW-to-population ratio in Africa stands at approximately 0.6 per 1000 people,^14^ far below the recommended levels necessary to achieve UHC and sustainable development goals (SDGs). Despite the recognised need for the services of CHWs, their integration into the PHC system remains slow mainly because of inadequate support at the policy level.^30^ The inclusion of oral healthcare into the roles and responsibilities of CHWs can only stem from the integration of oral health into PHC delivery. Efforts in this respect are ongoing but slow, further limiting the possibility of CHWs to promote oral health.

Country-level policies are needed to outline CHWs’ roles and responsibilities for promoting oral health and to establish standardised training programmes. The governance structures for CHWs should facilitate coordination across stakeholders, including governments, donors and community organisations, to ensure that CHWs are adequately supported to play their role in educating and referring community members for oral healthcare. Research is also needed to learn about effective, feasible and acceptable CHW oral health programmes that can serve as the foundation for policies that are responsive to local needs. An effective CHW programme must align global strategies with local scenarios and be flexible enough to allow for context-specific adaptation to reflect the realities of local health systems, cultural norms and community needs.^31^ Engaging communities and CHWs themselves in the design and governance of these programmes fosters ownership, ensures cultural acceptability and improves sustainability.

### Resource constraints

Community health worker programmes face severe underfunding, with a $5.4 billion annual funding gap.^31^ This shortfall is largely because of disease-focussed programming, where resources are disproportionately allocated to high-profile diseases such as malaria, tuberculosis and HIV and/or acquired immunodeficiency syndrome (AIDS).^1,32^ This narrow focus leads to inefficient investments, leaving other critical areas such as oral health underfunded and underserved. The lack of funds undermines the effectiveness of CHW programmes, impacting the quality and consistency of service delivery. For oral health programmes, this means limited access to essential supplies such as fluoride varnish, toothbrushes and educational materials, hindering comprehensive care.

Evidence shows the lack of government prioritisation, where oral health often ranks lower than other health concerns, affecting both domestic and international funding. This was affirmed by a study which reported that in 2019, most African countries spent less than $1.00 per capita on dental care.^33^ Donors typically fund initiatives that align with global health priorities, further reducing the support for oral health services provided by CHWs. This lack of prioritisation and funding from both governments and external donors severely constrains the ability of CHWs to deliver essential oral health services.

## Strengthen the integration of oral health into primary healthcare

The literature shows that the integration of CHW programmes into the formal health system is crucial for delivering consistent and quality oral health services.^34^ This integration ensures better coordination, supervision and support, addressing the limitations of siloed CHW programmes. Embedding CHWs within oral PHC centres or district health offices makes them part of the oral healthcare team, facilitating better communication and coordination with other providers.^35,36,37^ This can allow CHWs to access and contribute to unified health records, ensuring consistent and updated oral health information. Integration also establishes formal referral processes and follow-up mechanisms, ensuring patients receive appropriate and continued care. It also helps track patient progress and identify gaps in care.

Regular supervision within the formal healthcare system is needed to ensure that CHWs adhere to standardised protocols and receive feedback to improve performance.^38^ Integrating CHW programmes into the formal health system should emphasise a systems-thinking approach, which highlights the interdependence and communication between different healthcare components to ensure holistic patient care.

Integration is also cost-effective, as it optimises resource use by reducing redundancies, improving service delivery efficiency and leveraging the existing healthcare systems infrastructure to support CHWs.^39^ This can minimise the costs associated with fragmented programmes and maximise the return on investment in CHW programmes by ensuring that their efforts are aligned with broader health system objectives. Ultimately, integration strengthens the ability of the overall health system to deliver equitable, accessible and high-quality oral healthcare to underserved populations.^40^

## Training and continuing education

To effectively include oral healthcare among CHW responsibilities, CHWs need specific training, as their education typically focusses on general health. Even short training sessions can significantly improve CHWs’ ability to recognise oral health issues.^9^ Dental schools in Africa represent an underutilised yet highly strategic resource for strengthening oral health programmes for CHWs. Dental schools can strengthen CHW programmes by developing locally relevant, evidence-based training curricula that address community-specific oral health needs. They can provide hands-on training for procedures such as screenings and minor infection management, by using simulations and supervised placements to build CHWs’ confidence, and act as hubs for continuous education, offering refresher courses and certifications. This collaboration enables career advancement for CHWs and addresses workforce shortages. By integrating CHWs into interprofessional teams, dental schools can bridge the gap between community-level interventions and clinical care, promoting a holistic approach to oral health.

Structured training programmes, continuing education and essential resources enhance the competence and confidence of CHWs in delivering quality oral health services. By being embedded in the formal health system, CHWs can participate in capacity-building initiatives that align with national health goals, improving both service delivery and workforce sustainability.

## Research, monitoring and evaluation

While various CHW projects in Africa show promise in delivering oral healthcare, the absence of a national, integrated programme limits their scalability and impact. Establishing a national CHW programme that incorporates oral healthcare, guided by evidence from implementation science, is essential for addressing the high burden of oral diseases and improving public health outcomes across Africa. Dental schools can contribute to this goal by conducting research on CHW oral health programmes. They can also evaluate the effectiveness of various training methods and tools, ensuring that the programmes are evidence-based.

Implementing robust monitoring and evaluation systems is crucial for improving oral health initiatives.^41^ These systems provide a framework for assessing programme performance, ensuring accountability and driving continuous improvement. Key components include clear performance indicators, regular audits to evaluate service quality and resource use, and actionable feedback mechanisms to refine practices. Integrating technology, such as mobile apps and GIS, enhances real-time data collection and resource allocation. Monitoring and evaluation systems also support sustainability by generating evidence to guide policy and funding decisions, ensuring that programmes remain relevant and impactful for evolving community needs.^42^

## Role of the Africa Centres for Disease Control and Prevention in community health worker programmes for oral health in Africa

It is important to capitalise on the Africa Centres for Disease Control and Prevention’s (CDCs) CHW programme,^43^ by integrating oral health into community health responses from the programme’s inception to enhance public oral health outcomes. Epidemics and pandemics often worsen oral health by limiting access to care, which negatively impacts overall health and quality of life.^44^ To achieve UHC and ensure health security in Africa, CHW programmes must recognise and address oral health as a public health priority.

The Africa CDC should work with national health ministries to create policies that clearly define the role of CHWs in promoting oral health, providing preventive care and referring patients to specialised care. The programme should integrate cost-effective oral health initiatives funded by domestic resources into CHW programmes during both peacetime and pandemic responses to improve health.^45,46,47^ The Africa CDC advocated for local vaccine production because of the devastating effects of coronavirus disease 2019 (COVID-19) and mpox on the lives and well-being of Africans despite the availability of vaccines in the Global North.^48^ Similarly, strategies should be developed to locally produce basic oral health products such as fluoride gels, glass ionomers and affordable toothpastes so that they can be used in the CHW programme.

The Africa CDC has the potential to significantly enhance access to oral health in Africa through CHW programmes, but several challenges could impede its efforts. These include insufficient funding, weak integration of oral health into national health strategies, inconsistent CHW training curricula, poor healthcare infrastructure, limited data for policymaking and uneven political commitment across member states.

To overcome these barriers, the Africa CDC can advocate for increased funding and support the integration of oral health into national public health policies. Standardising CHW training materials tailored to local contexts, strengthening data collection systems and fostering regional collaboration will enhance programme effectiveness. By leveraging its influence, the Africa CDC can build political commitment and coordinate multi-sectoral efforts, ensuring that oral health becomes a core component of health systems across the continent.

## Conclusion

Expanding the CHW programmes in Africa to include oral health promotes a holistic approach to health. Addressing oral health issues can prevent complications related to systemic conditions and reduce the burden of oral diseases and their associated healthcare costs. In addition, CHWs are trusted community members, and their expanded role in oral healthcare can increase community engagement and utilisation of oral healthcare services. Community health workers can promote oral health in Africa, bridging the gap between communities and formal healthcare systems. Continuing training, efficient resource allocation, policy development and system integration will empower CHWs to make a significant impact on oral health outcomes. Collaborative efforts from all stakeholders are crucial to maximise the potential of CHW programmes and move closer to achieving UHC and improved public health in Africa. A comprehensive assessment of the status of the integration of oral health into CHW programmes in Africa is needed. This can help the Africa CDC identify gaps for strategic actions.
